# Implantable cardiac defibrillator outcomes in octogenarians

**DOI:** 10.1002/joa3.70012

**Published:** 2025-02-13

**Authors:** Bryan Stringer, Luke MacLeod, Fady Kaldas, Gayuni Krishnasamy, Habib Rehman Khan

**Affiliations:** ^1^ Western University Schulich School of Medicine and Dentistry London Ontario Canada

**Keywords:** frailty, implantable cardiac defibrillator, octogenarians, primary prevention, sudden cardiac death

## Abstract

**Background:**

Implantable cardiac defibrillators (ICDs) are essential for preventing sudden cardiac death. Despite inclusion in national guidelines, older adults are often underrepresented in trials. Evaluating ICD use in the aging population, particularly with advancements in heart failure treatment, is crucial.

**Objective:**

This study examines outcomes in octogenarians undergoing ICD implantation for primary and secondary prevention, focusing on ICD therapies and mortality timing.

**Methods:**

A retrospective observational study at a single Canadian academic center included patients ≥80 years old at ICD implantation, excluding those with <30 days follow‐up. Data on demographics, comorbidities, mortality, and ICD therapies were collected from electronic medical records. Clinical frailty was assessed using the Dalhousie Clinical Frailty Scale.

**Results:**

We identified 143 patients (mean age 82.6 ± 2.2 years, 14% female) from May 2015 to October 2023. ICDs were implanted for primary prevention in 63 patients (44%) and secondary prevention in 80 patients (56%). Thirty‐seven patients were excluded due to insufficient follow‐up. ICD therapies occurred in 30 patients (25%) through anti‐tachycardia pacing (ATP) and in 19 patients (18%) through shocks. The mean time to first ATP was 16.9 ± 21.0 months, and to first shock, 21.2 ± 23.6 months. Among 66 patients with mortality data, 19 (24%) died at 31.3 ± 30.4 months. Patients with non‐ischemic cardiomyopathy experienced earlier shocks (7.7 vs. 32.2 months, *p* < 0.05).

**Conclusion:**

Elderly patients undergoing ICD implantation have multiple comorbidities and competing causes of mortality. Device use is overall infrequent but occurs well before observed mortality. Prospective clinical trials are needed to determine ICD benefits in this age cohort.

## Graphical abstract

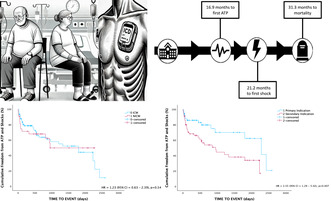

## 1 INTRODUCTION

Implantable cardiac defibrillators (ICDs) play a crucial role in both primary and secondary prevention of sudden cardiac death (SCD). The guidelines for ICD indications were delineated in the 2016 edition of the Canadian Cardiovascular Society/Canadian Heart Rhythm Society Guidelines.^1^ Primary prevention data is drawn from numerous randomized trials.^2^, ^3^, ^4^, ^5^, ^6^ However, recent studies have presented conflicting findings regarding the efficacy of ICDs in patients with non‐ischemic cardiomyopathy (NICM), particularly among those aged over 70 years.^7^ Notably, these studies often underrepresent or exclude octogenarians.

A recent meta‐analysis combined data from five major clinical trials, focusing on patients aged over 75 years. While older patients exhibited increased mortality, they still experienced a reduction in mortality risk with ICD use. Notably, only about 2.8% of the 3530 patients in this meta‐analysis were octogenarians.^8^

However, this percentage doesn't accurately reflect the rate of device implantation in this age group. Registry data from 2009 indicated that over 40% of new ICDs and CRT‐Ds were implanted in patients aged over 70, with over 10% in those aged 80 and above.^9^ Given the data's age, it's reasonable to assume that implantation rates in this demographic have risen since then. Retrospective studies conducted between 2015 and 2023 also corroborate this trend, with ICD implant percentages in patients over 80 years old ranging from 10% to 20%.^10^, ^11^, ^12^, ^13^, ^14^

As the global population ages, the prevalence of cardiomyopathy and arrhythmias within this demographic is expected to escalate. However, implanting an ICD in older patients is not without challenges, as they face higher mortality and complication rates, potentially impacting their quality of life or leading to end‐of‐life complications.^15^ Given these inherent risks and the lack of robust trial data in this population, shared decision‐making with older patients becomes imperative.

Our study aims to analyze outcomes in octogenarians with ICDs, focusing on device utilization and mortality. Recognizing that individuals aged 65 and older are more prone to frailty, which can influence their risk of adverse health outcomes, we also intend to assess patient frailty and its impact on outcomes.

## 2 METHODS

### 2.1 Patient population

This was a retrospective, observational study at a single academic center in Canada. The electronic medical record was utilized to query procedural codes to identify patients who underwent implantation of any ICD device from May 2015 to October 2023. Patients who were ≥80 years old at ICD implantation were included. To be in the primary analysis defined below, patients required at least 30 days of follow‐up after implanting within the device clinic. This requirement was to ensure that only patients with sufficient device‐related data were included in the primary analysis as our center implants cardiac devices for a large catchment area but does not routinely provide their follow up. Follow is often at remote sites and device‐related data is not readily accessible. Patient devices had standard programming. For primary prevention, the ventricular tachycardia (VT)/ventricular fibrillation (VF) zone was set at 188 beats per minute (bpm) with anti‐tachycardia pacing (ATP) while charging, followed by 35J shocks. In patients with a secondary prevention indication, if the heart rate was >188 bpm it would follow the primary prevention programming. If the heart rate was <188 bpm, the VT zone was set at 150–188 bpm with ATP burst 8 pulses at 88% for 3 sequences, followed by RAMP 91% for 12 pulses, then 4 shocks at 35J. VT monitor zone was 130–150 bpm.

### 2.2 Data collection

Data was obtained from the patient's electronic medical record and ICD clinic charts. This included patient demographics, comorbidities, prescription medications and echocardiography data. A frailty assessment was performed using the Dalhousie Clinical Frailty Score (CFS), which has been applied retrospectively in previous studies.^16^ Based on this score (1–3, 4–6 and 7–9, respectively), frailty was stratified into mild, moderate, and severe. ICD clinic data was queried to obtain information on device activation and therapies, including ATP and shocks. Lastly, the electronic medical record was assessed for mortality information. If patients had documentation of any clinic appointment, emergency department visit or inpatient note within 3 months of the study end date (November 2023), they were presumed to be alive.

### 2.3 Outcomes and sub‐group analysis

The study's primary outcome was the time from implant to first therapy (ATP or shocks) measured in months. Secondary outcomes included the total amount of therapies and mortality.

Sub‐group analysis included comparing patients with primary and secondary indications for ICD, ischemic cardiomyopathy (ICM) and NICM, and patients with varying frailty severity.

### 2.4 Statistical analysis

A Student's t‐test or one‐way ANOVA was used for continuous data. A Wald's test was performed to compare proportions among different cohorts.

Ethics approval for this study was obtained from the Research Ethics Board at Western & Lawson research.

## 3 RESULTS

A total of 143 patients were identified between May 2015 and October 2023 to have received an ICD. Among these patients, 20 were female (14%), and the mean age at the time of implantation was 82.6 ± 2.2 years. Primary prevention was the indication for an ICD in 62 patients (43%), while the remainder received ICDs for secondary prevention against VT, VF, and sudden cardiac arrest (SCA) where rhythm strip was not available, but the patient was resuscitated. Most patients had comorbid hypertension (73.4%) and congestive heart failure (HF) (CHF) (92.3%). Additional baseline characteristics of the patients are summarized in Table 1.

**TABLE 1:** Patient baseline characteristics.

Parameters	All patients (*n* = 143)
Age (years) mean ± SD	82.6 ± 2.2
Female, *n* (%)	20 (14)
Indication, *n* (%)	
Primary prevention	62 (43.4)
VT	62 (43.4)
VF	14 (9.7)
SCA	5 (3.5)
Hypertension, *n* (%)	105 (73.4)
Diabetes, *n* (%)	45 (31.5)
Atrial Fibrillation, *n* (%)	71 (49.7)
Concurrent pacemaker indication, *n* (%)	35 (24.5)
Cardiac resynchronization therapy at time of implant, *n* (%)	28 (19.5)
Congestive heart failure, *n* (%)	132 (92.3)
Ischemic cardiomyopathy, *n* (%)	96 (67.1)
Non‐Ischemic cardiomyopathy, *n* (%)	36 (25.2)
Undetermined etiology, *n* (%)	11 (7.7)
MCI/Dementia, *n* (%)	4 (2.8)
Pre‐implant LVEF (%), mean ± SD	36.1 ± 12.6
Antiarrhythmic medication, *n* (%)	49 (34.3)
Beta blockers, *n* (%)	117 (81.8)
Clinical Frailty Score, mean ± SD	3.5 ± 1.8
Mild (1–3)	50
Moderate (4–6)	24
Severe (7–9)	5

*Note*: Values are expressed as mean ± standard deviation.

Abbreviations: LVEF, left ventricular ejection fraction; MCI, mild cognitive impairment; SCA, sudden cardiac arrest; VF, ventricular fibrillation; VT, ventricular tachycardia.

**FIGURE 1:**
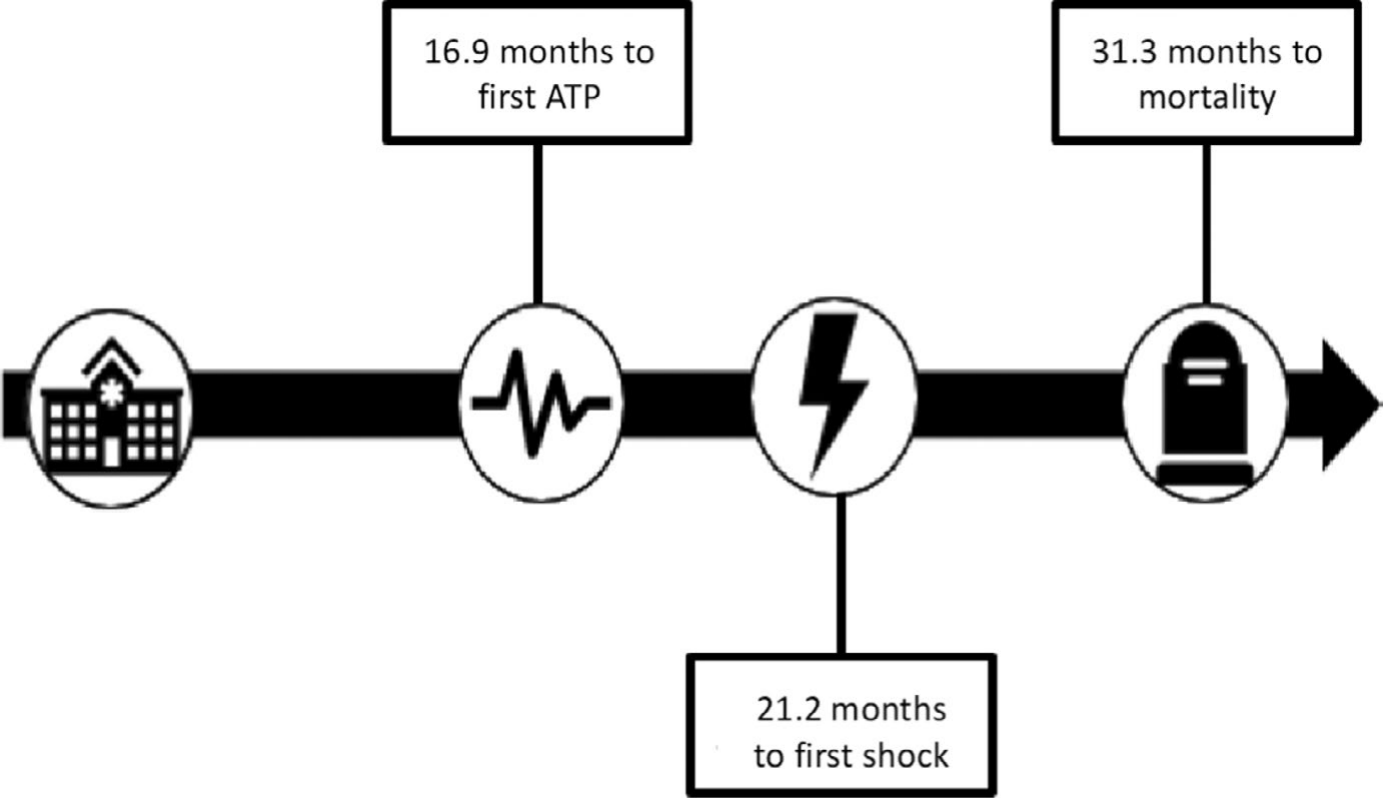
Timeline from device implantation to mortality. Values expressed are the mean. ATP, anti‐tachycardia pacing.

Of the 143 patients, 37 were excluded from the primary analysis due to missing ICD therapy data due to remote ICD clinic follow‐up where the referring center failed to provide data. Among those included, the mean follow‐up duration in the device clinic was 30.1 ± 26.5 months (Table 2). ATP occurred in 30 patients (25%), with the first therapy occurring on average 16.9 ± 21.0 months after device implantation. Nineteen patients experienced a device shock (18%) at an average of 21.2 ± 23.6 months. Of these 19, only 1 patient (5.3%) experienced an inappropriate shock for external noise. Among the 80 patients with sufficient data for mortality assessment, 19 (24%) had died, with a mean time to mortality of 31.3 ± 30.4 months from device implantation (Figure 1). Of these 19 patients, 18 were able to have a cause of death determined. Fifty percent of patients died from a cardiac etiology. This was mostly due to worsening heart failure and cardiogenic shock. There was one case of a patient having refractory ventricular arrhythmias and their device was deactivated as part of a transition to palliative care. The remainder included death from worsening renal failure, lung pathology, or malignancy.

**TABLE 2:** Primary analysis of device therapy and time to therapy.

Patients with adequate follow up	106
Follow‐up duration (month)	30.1 ± 26.5
ATP	30 (25%)
Shock	19 (18%)
Time to first ATP (month)	16.9 ± 21.0
Time to first Shock (month)	21.2 ± 23.6
Total mortality (*n* = 80)	19 (24%)
Time to mortality (month)	31.3 ± 30.4

Abbreviation: ATP, anti‐tachycardia pacing.

**FIGURE 2:**
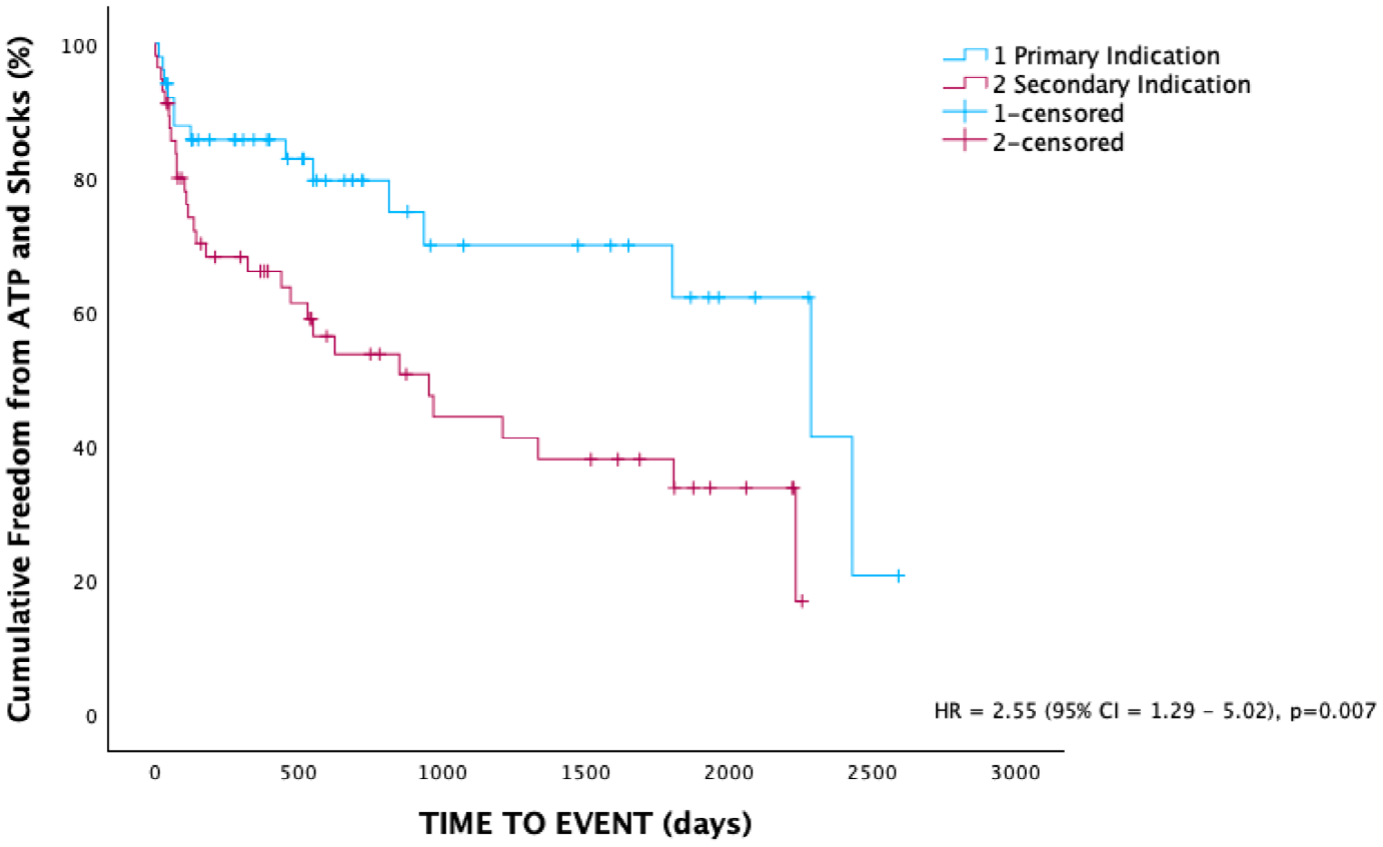
Cumulative freedom from ATP or shocks in patients with primary versus secondary prevention ICD indications. ATP, anti‐tachycardia pacing; ICD, implantable cardiac defibrillator.

Patients with primary prevention ICDs had a significantly lower baseline ejection fraction than those with secondary prevention ICDs (28.8 ± 6.3% vs. 41.0 ± 13.4%, *p* < 0.001). A higher proportion of patients with secondary prevention indication experienced ATP (39.2% vs. 16%, *p* < 0.05). Over the follow‐up period, patients with a primary prevention ICD had significantly higher cumulative freedom of any device therapy compared to secondary prevention (Figure 2). There was no significant difference in the proportion of patients experiencing shocks (23.2% vs. 12%, *p* = 0.13). There was no significant difference in time to initial ATP or shocks. Similarly, there was no difference in the time to mortality between the two groups (Table 3).

**TABLE 3:** Comparison of primary versus secondary prevention indications.

Baseline	Primary prevention (*n* = 62)	Secondary prevention (*n* = 81)	*p* Value
Age (years) mean ± SD	82.3 ± 2.0	82.0 ± 7.0	0.10
Pre‐implant LVEF (%) mean ± SD	28.9 ± 6.3	41.0 ± 13.4	<0.001
Adequate device follow‐up (*n*)	50	56	
ATP, *n* (%)	8 (16)	22 (39.2)	<0.05
Shock, *n* (%)	6 (12)	13 (23.2)	0.13
Any therapy, *n* (%)	9 (18)	24 (42.9)	<0.05
Time to first ATP (month) mean (95% CI)	19.6 (2.2–36.0)	15.6 (6.1–25.1)	0.65
Time to first Shock (month) mean (95% CI)	17.5 (0.5–41.6)	22.5 (7.8–37.1)	0.68
Time to mortality (month) mean (95% CI)	39.6 (30.3–109.4)	29.5 (12.4–46.7)	0.59

Abbreviations: ATP, anti‐tachycardia pacing; LVEF, left ventricular ejection fraction.

**FIGURE 3:**
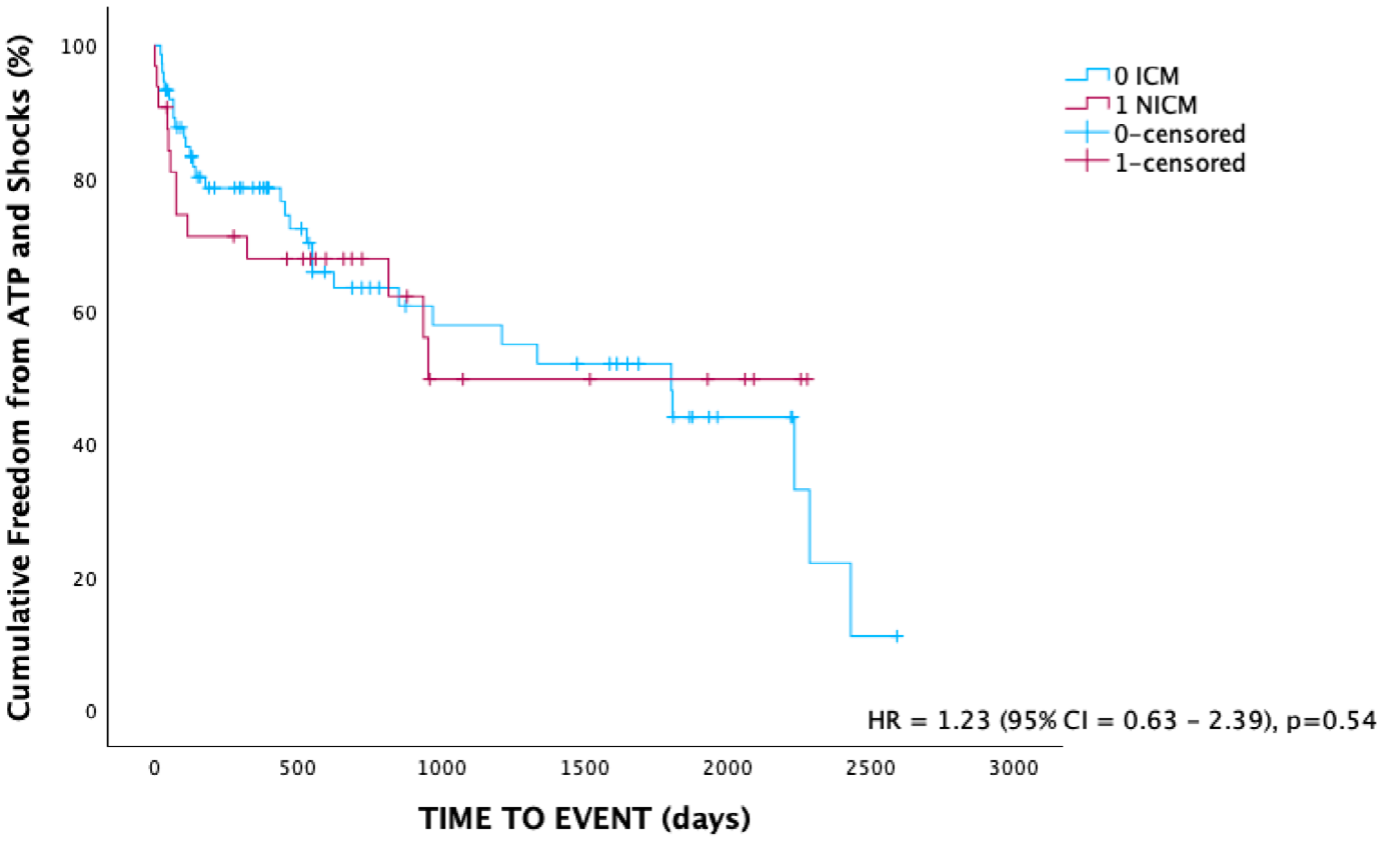
Cumulative freedom from ATP or shocks in patients with ischemic versus non‐ischemic cardiomyopathy. There was no significant difference between the two groups. ATP, anti‐tachycardia pacing; ICD, implantable cardiac defibrillator.

There was no significant difference in baseline ejection fraction between ICM and NICM patients (Table 4). There was no difference in the incidence of ATP use between these groups, nor was there a significant difference in the number of shocks (Table 5). Over the follow‐up period, there was no significant difference in cumulative freedom from device therapy between the two groups (Figure 3). Patients with ICM received shocks much later than NICM (32.2 vs. 7.7 months, *p* < 0.05). There was no significant difference in time to mortality. Of those with a documented cause of their NICM, majority were labeled as a dilated cardiomyopathy. There was 1 patient with Fabry's disease, 1 stress‐induced cardiomyopathy, 1 tachycardia mediated cardiomyopathy and 2 cases of ATTR amyloid cardiomyopathy. A total of 11 patients had an undetermined etiology of their cardiomyopathy.

**TABLE 4:** Comparison of ischemic cardiomyopathy versus non‐ischemic cardiomyopathy. There were 11 patients with undetermined etiology of cardiomyopathy.

Baseline	NICM	ICM	*p* Value
	(*n* = 36)	(*n* = 96)	
Age (years)	81.9 ± 1.69	82.1 ± 2.37	0.08
Pre‐implant LVEF (%)	35.8 ± 12.6	33.6 ± 11.5	0.36
Adequate device follow up	28	68	
ATP, *n* (%)	8 (28.6)	19 (27.9)	0.59
Shock, *n* (%)	7 (25)	10 (14.7)	0.22
Any therapy, *n* (%)	10 (35.7)	20 (29.4)	0.55
Time to first ATP (month) mean (95% CI)	8.2 (4.5–18.9)	21.7 (10.5–32.9)	0.14
Time to first Shock (month) mean (95% CI)	7.7 (4.2–17.7)	32.2 (16.6–51.5)	0.02
Time to mortality (month) mean (95% CI)	37.6 (15.4–59.9)	16.5 (15.1–91.3)	0.40

Abbreviations: ATP, anti‐tachycardia pacing; LVEF, left ventricular ejection fraction.

**TABLE 5:** Comparison of ATP use in patients with ischemic cardiomyopathy versus non‐ischemic cardiomyopathy.

ATP amount	ICM	NICM	*p* Value
	(*n* = 19)	(*n* = 8)	
Mean	29.4 ± 35.3	59.5 ± 62.8	0.12
<10	7 (36.8)	2 (25)	0.55
10–49	5 (26.3)	2 (25)	0.94
50–100	3 (15.8)	2 (25)	0.57
>100	1 (5.3)	2 (25)	0.14

Abbreviation: ATP, anti‐tachycardia pacing.

Seventy‐nine patients had sufficient retrospective data to estimate a Dalhousie Frailty score. Among them, 50 patients (63.3%) had scores indicating mild frailty (1–3), 24 (30.4%) had scores indicating moderate frailty (4–6), and 5 (6.3%) had scores indicating severe frailty (7–9). As frailty increased, there was a decrease in the proportion of patients with primary prevention ICDs, with 42%, 37.5%, and 20% in the mild, moderate, and severe frailty groups, respectively. Among those with mild frailty, 17.5% experienced shocks, significantly fewer than those with moderate and severe frailty (both at 25%, *p* < 0.05). However, there was no significant difference in time to initial shock. There was no significant difference in the proportion of patients requiring ATP among the different frailty cohorts (*p* = 0.15). Furthermore, there was no significant difference in mortality (*p* = 0.6804).

In 143 patients undergoing device implant, 6 patients (4.2%) required an additional procedure. Three patients required a lead reposition/revision for either low thresholds or lead dislodgement. One patient required both atrial lead revision and pocket revision for skin erosion. One patient required pocket revision for expanding hematoma. One patient needed device extraction due to infection. In addition to patients who had an unanticipated repeat procedure, an additional 5 patients (3.5%) went on to have an upgrade to a CRT device.

## 4 DISCUSSION

This retrospective study evaluated the outcomes of octogenarians who received ICDs over an 8‐year period. Our findings contribute to the expanding body of retrospective studies providing valuable insights into the clinical effectiveness of ICDs in elderly patients, particularly concerning device therapies, mortality, and the influence of frailty.

As the median age of cardiac patients increases, there is a notable increase in ICD implantations among the elderly population, particularly those aged over 70 years.^17^, ^18^ This population frequently presents with a history of heart failure (HF) and myocardial infarction, thus emphasizing the role of ICDs in their management. However, the current guidelines for ICD placement in octogenarians are ambiguous, as landmark ICD trials have excluded or underrepresented patients over the age of 80, leading to inadequate representation in clinical guidelines.^19^, ^20^ As medical therapy for HF improves, high‐quality evidence is urgently needed to guide decision‐making in this patient group.^21^

In our study, approximately 25% of the population experienced ATP, and 18% received shocks, with a mean time to the first therapy of about 17 months post ICD implantation. These results are consistent with prior studies in older populations, indicating that ICDs remain effective in terminating life‐threatening arrhythmias even in advanced age groups.^12^, ^14^ Interestingly, we found no significant difference in the incidence of shocks between patients receiving ICDs for primary versus secondary prevention (12% vs. 23.2%, respectively, *p* = 0.13). However, secondary prevention patients were more likely to experience ATP, which may reflect a higher arrhythmic burden in this group. This observation aligns with previous studies demonstrating a 74% increased risk of appropriate ICD therapies in the secondary prevention groups compared to primary prevention.^22^

Mortality outcomes in older patients undergoing ICD implantation have varied across studies. In a nationwide registry from Belgium, authors report a mortality rate of 35% in a mean follow‐up of 3.1 years, in which 11% of these patients died within the first year.^11^ Another European study reported even higher mortality rates, with 89% of octogenarians dying within 3.3 years of ICD implantation or generator change.^10^ In contrast, our study's mortality rate of 24% over a mean follow‐up of 31 months is comparable to rates reported in studies from the UK and Australia.^12^, ^13^ In the Australian population, authors reported a 20% mortality rate after 2 years following ICD implant, though their cohort had a median Charlson Comorbidity Index score of 0, indicating a healthy population.^13^ Our study population, with significant comorbidities such as HF and hypertension, demonstrated mortality consistent with expectations for older individuals. Notably, 50% of the deaths were attributable to cardiac causes, highlighting the ongoing risk of cardiovascular mortality despite ICD therapies. Non‐cardiac causes of death, including renal failure and malignancies, highlight the competing risks faced by this frail population, reinforcing the need for comprehensive patient management in this age group.

The benefits of ICDs in secondary prevention across all populations are well established. However, the benefits of primary prevention ICDs in elderly patients remain unclear. A meta‐analysis of five clinical trials demonstrated that the mortality benefit of ICD therapy was preserved throughout the age spectrum, specifically in patients over 75 years old.^8^ Nevertheless, only 11% of the trial participants were over 75, and less than 3% were over 80. Furthermore, these findings predated the DANISH trial, which assessed ICD use in NICM.^23^ The 10‐year extended follow‐up of the DANISH trial demonstrated no difference in all‐cause mortality in patients over 70 treated with ICDs versus usual clinical care.^7^ Despite these findings, current guidelines do not differentiate ICD recommendations based on age; instead, they focus on life expectancy of at least 1 year to be considered for ICD placement.^24^, ^25^

The individualized decision for ICD implantation in elderly patients is complex and nuanced, especially in the presence of multiple comorbidities and competing mortality risks. One comorbidity often overlooked in studies and trials is frailty. Our study retrospectively calculated the Dalhousie CFS, a validated tool for assessing frailty in elderly cardiovascular patients. The CFS score ranges from 1 (very fit) to 9 (terminally ill), with higher scores indicating greater frailty. Most patients exhibited only mild frailty, with only 6% classified as highly frailty. We found that frailty was inversely associated with the likelihood of receiving ICDs for primary prevention, and patients with moderate to severe frailty experienced higher shock rates. This raises concerns about the appropriateness of ICD therapy for frail patients, who may derive less benefit due to competing risks of non‐cardiac mortality and limited overall prognosis. However, there was no significant difference in the time to initial shock or ATP across the frailty spectrum, suggesting that frailty affects the likelihood but not the timing of shocks. A recent systematic review confirmed that frailty is associated with increased mortality after ICD placement, using both single‐marker frailty indices and multi‐domain instruments.^26^ Thus, frailty should be considered a critical factor in shared decision‐making influencing morbidity and mortality outcomes.

### 4.1 Limitations

This study is limited by its retrospective, single‐center design, which prevents conclusions about causality or mortality benefits. Additionally, a significant portion of our patients were lost to follow‐up, with over one‐third of patients having their device clinic data recorded at peripheral sites, rendering therapy data inaccessible. The study's design, which included only patients with at least 30 days of follow‐up, may have excluded patients with early complications or mortality shortly after the device implantation. These limitations highlight the need for caution when interpreting the study's findings and emphasize the necessity of future randomized clinical trials to address these constraints.

### 4.2 Future direction

Significant uncertainty remains surrounding ICD implantation in elderly patients. Given the complexity of this patient cohort characterized by a high incidence of comorbidities such as frailty and varying patient values, a one‐size‐fits‐all approach is unlikely to be appropriate. Instead, treatment decisions need to be individualized. A randomized clinical trial is urgently needed to assess the mortality benefit of ICDs in this population. Recently, the I‐70 study was published, which attempted to address this question in veterans over the age of 70. However, only 11% of the sample size target was achieved, and the study was halted due to recruitment challenges.^27^ This highlights the challenges of enrolling patients in such trials and suggests that a large, multi‐center, international study will be needed to address this issue adequately.

## 5 CONCLUSIONS

This study adds to the growing literature on ICD implantation in elderly patients despite a paucity of prospective evidence of their benefit. It further demonstrates that octogenarians often have multiple comorbidities, including frailty, and have competing causes of mortality. Nonetheless, ICDs are widely utilized, and their use is associated with decreased arrhythmic death. However, a randomized clinical trial is needed to determine if this is indeed the case in the octogenarian population.

## CONFLICT OF INTEREST STATEMENT

None of the authors have any conflict of interest.

## ETHICS STATEMENT

This original article complies with the journal's ethics and integrity policies. Ethics approval for this study was obtained from the Research Ethics Board at Western & Lawson research. The research ethics board waived the need for patient consent due to the retrospective nature of this study. This study is not part of a clinical trial or registry.

## Data Availability

Data is available for sharing upon request.
